# Cryptic severe *Plasmodium falciparum* malaria in a Moroccan man living in Tuscany, Italy, August 2018

**DOI:** 10.2807/1560-7917.ES.2018.23.41.1800527

**Published:** 2018-10-11

**Authors:** Lorenzo Zammarchi, Nicoletta Di Lauria, Filippo Bartalesi, Lorenzo Roberto Suardi, Giampaolo Corti, Jessica Mencarini, Daniela Boccolini, Carlo Severini, Luigi Gradoni, Carla Buonamici, Giorgio Garofalo, Anna Maria Bartolesi, Nunziata Ciccone, Andrea Berni, Loredana Poggesi, Fabrizio Niccolini, Gian Maria Rossolini, Roberto Romi, Alessandro Bartoloni

**Affiliations:** 1 Department of Experimental and Clinical Medicine, University of Florence, Florence, Italy; 2 Infectious and Tropical Diseases Unit, Careggi University Hospital, Florence, Italy; 3 Department of Infectious Diseases, National Institute of Health, Rome, Italy; 4 Public Health Department, USL Toscana Centro, Italy; 5 Microbiology and Virology Unit, Careggi University Hospital, Florence, Italy; 6 Internal Medicine Unit 3, Careggi University Hospital, Florence, Italy; 7 Health Direction, Careggi University Hospital, Florence, Italy

**Keywords:** malaria, Plasmodium falciparum, autochthonous, Anopheles spp., Italy, Europe

## Abstract

In August 2018 a Moroccan man living in Tuscany developed *Plasmodium falciparum* malaria. The patient declared having not recently visited any endemic country, leading to diagnostic delay and severe malaria. As susceptibility to *P. falciparum* of Anopheles species in Tuscany is very low, and other risk factors for acquiring malaria could not be completely excluded, the case remains cryptic, similar to other *P. falciparum* malaria cases previously reported in African individuals living in Apulia in 2017.

A case of severe *Plasmodium* *falciparum* malaria was diagnosed in August 2018 in a Moroccan man living in Tuscany, Italy. Malaria was initially not considered by the managing clinicians because the patient reported to have not recently visited any endemic country, leading to diagnostic delay and severe anaemia. Here we describe the clinical and epidemiological features of this case and discuss different hypotheses on the possible route of transmission.

## Case report

A previously healthy Moroccan man in his late 40s was admitted to an internal medicine ward of a tertiary hospital in Florence, Italy, in mid-August 2018, with a 3-day history of high fever (> 38.5°C), headache, and mild cough. At physical examination, jaundice and splenomegaly were observed. Blood tests showed thrombocytopenia, haemolytic anaemia, and increased inflammatory markers (Table). A haematological disease was suspected, while malaria was initially not considered, since the patient declared to have not recently visited any endemic area. On Day 4 after admission, investigation for malaria was requested following an infectious diseases consultation. Thin blood smears revealed the presence of *P. falciparum* trophozoites with a parasitaemia of 0.5%. PCR confirmed *P. falciparum* mono-infection. The patient was referred to the Infectious and Tropical Diseases Unit with the diagnosis of severe malaria (haemoglobin (Hb) < 7 g/dL and parasitaemia > 0.2% [1]), and intravenous artesunate was administered for 2 days, followed by oral dihydroartemisin-piperaquine for 3 days. Two units of packed red blood cells were transfused. The patient's conditions improved and he was discharged a few days later. The case was notified to public health authorities (malaria is a mandatory notifiable disease in Italy) and microscopically confirmed by the National Institute of Health, Rome, Italy [2]. 

**Table:** Summary of main blood and urine investigations performed in the patient with *Plasmodium falciparum* malaria, Tuscany, Italy, August 2018

Parameters	Time after admission (Day 1) at the hospital in August 2018					
	Day 1	Day 2	Day 3	Day 4	Day 5	Day 6
White blood cells (x10^9/L) (norm: 4.00–10.00)	6.19	4.31	4.87	3.01	3.91	4.51
Haemoglobin (g/dL) (norm: 14–18)	12.1	10.5	9.7	6.9	8.9	8.8
Haematocrit (%) (norm: 42.0–52.0)	34.6	30	27.7	19.9	25.7	25.9
Platelet count (x10^9/L) (norm: 14–440)	65	44	70	79	101	261
Prothrombin time (%)(norm: 70–130)	62	61	69	70	ND	ND
INR (norm: 0.8–1.2)	1.4	1.4	1.3	1.3	ND	ND
Partial thromboplastin time (sec) (norm: 22–38)	27.1	27.5	27.7	27.1	ND	ND
Fibrinogen (mg/dL) (norm: 200–400)	597	668	709	462	ND	ND
Glucose (mg/dL) (norm: 65–110)	117	102	105	ND	ND	82
Creatinine (mg/dL) (norm: 0.44–0.9 )	0.92	0.93	0.97	0.91	0.77	0.69
Sodium (mEq/L) (norm: 135–145)	135	133	134	136	139	139
Potassium (mEq/L) (norm: 3.5–5)	ND	3.4	3.8	3.6	4	3.9
Alanine aminotransferase (U/L) (norm: 12–65)	53	40	ND	ND	40	18
Total bilirubin (mg/dL) (norm: 0.2–1)	3.2	3.1	4.2	2.0	ND	ND
LDH (U/L) (norm: 84–246)	ND	640	866	ND	733	ND
Haptoglobin (g/L) (norm: 0.3–2.0)	ND	< 0.078	ND	ND	ND	ND
C-reactive protein (mg/L) (norm: <9)	148	ND	ND	ND	88	ND
Procalcitonin (ng/mL) (norm: <0.5)	ND	ND	5.09	ND	1.20	ND
Reticulocyte count (%) (norm: 0.5–2.5)	ND	4.8	ND	ND	ND	ND
HIV 1–2 Ag/Ag (norm: neg)	ND	ND	ND	Neg	ND	ND
Urine screening test for drugs^a^ (norm: neg)	ND	ND	ND	Neg	ND	ND

Ag: antigen; HIV: human immunodeficiency virus; INR: international normalised ratio; LDH: lactate dehydrogenase; ND: not done; Neg: negative.

^a^ Including amfetamine, benzodiazepine, cocaine, ecstasy, marijuana, methadone, opiates.

## Epidemiological investigation

A careful history on possible malaria exposure was collected in collaboration with a Moroccan cultural mediator. The patient was born and had grown up in the Marrakesh-Safi Region, Morocco, before moving to Italy in 1990. He currently lives in an urbanised area of the province of Florence, Italy, about 10 km away from the city centre, where has been working, discontinuously, as a construction worker. He denied previous blood transfusions and intravenous drug use. He reported a 1-month travel to Ghana looking for a job in 2014. On that occasion, upon his arrival at Accra airport, he received an injection (probably, yellow fever vaccine) and was provided with some tablets (possibly antimalarial drug) that he took for about one week. During his stay in Ghana and thereafter, he did not present febrile diseases. He reported two to three travels to Morocco per year, to visit relatives in his village of origin for periods of up to 2 months. He returned from his last travel to Morocco, arriving by plane from Marrakech to Pisa in early June 2018, 73 days before the onset of symptoms. He slept one night in his residence near Florence, then moved by train to Viareggio, and subsequently worked outside as a beach vendor on the Tuscan coast during the summer. He stayed at the seaside and slept outdoors on the beach or in nearby places (for example in the pine forests along the coast, just behind the sand dunes), together with other beach vendors, including persons from Sub-Saharan Africa. The patient remembered many mosquito bites. He changed his location daily gradually moving south, and recalled having worked in beach resorts of Cecina, San Vincenzo, and Piombino, while he was not certain to have reached more southern beaches in the Maremma area. He returned to the starting point in Viareggio in mid-August. Then, he spent some time in a village near Bologna, and finally reached his home, 2 days later when he started to have fever in the afternoon. Over the whole summer, the patient worked and travelled with a group of newly-arrived men from Morocco who returned to their country on the day of fever onset in the patient; according to him all were in good health. No other information about them was available. 

## Discussion

Until the end of the 19th century, malaria was endemic in Italy, and the disease was responsible for ca 2 million cases and 15,000–20,000 deaths per year [3]. The most affected areas were the Southern and Central Regions (including the Maremma area in the Tuscany Region), Sicily, and Sardinia [3-5]. *P. falciparum* malaria accounted for ca 20–30% of cases in several regions including in the Maremma area of Tuscany [3]. At that time the proven mosquito vectors present in Italy were *Anopheles labranchiae*, *An. sacharovi*, and *An. superpictus* [6]. Several initiatives to control malaria and its vectors were implemented during the 20th century, including the reclamation of swamps, the extensive use of chemical pesticides, and the extensive delivery of quinine to the population [7]. These initiatives successfully led to a dramatic reduction of anopheline mosquitoes and to the elimination of the disease in 1970 [5]. Following the elimination, an increasing number of imported cases were reported in the country, in parallel to the rise of international travel and migratory flows, with ca 800 cases per year in the last two years [8].

An autochthonous introduced case of *P. vivax* malaria acquired in the Maremma area, Grosseto, Tuscany Region was recorded in August 1997 [9]. In that case, the source of the infection was identified to be a parasitemic Indian girl living about 500 m away from the patient’s residence, who had recently returned from a travel to her country of origin [9]. Several mosquito-breeding sites with *An. labranchiae* were identified during the entomological surveillance activities [9]. In separate events, another two potentially autochthonous introduced *P. vivax* malaria cases were diagnosed, one in August 2009 and one in September 2011, however their source of infection remained unidentified [10]. The patients had likely been infected in the Agro Pontino area, Latium Region (2009 case), and in the Cosenza province, Calabria Region (2011 case), respectively. Entomological investigations were able to detect the presence of *An. maculipennis* s.s. and *An. labranchiae* in the two episodes, respectively [10].

In October 2017, four *P. falciparum* malaria cases were reported in the Apulia Region, in three Moroccan and one Sudanese immigrants without recent history of travel outside Italy (some of them visited Morocco previously) [7,11]. All the affected patients were farm workers in the Ginosa or Castellaneta countryside, Taranto province [11,12], where the presence of *An. labranchiae* was confirmed by an entomological survey. All cases declared to have been in Italy for more than 3 months prior to onset of symptoms [11,13]. Based on these findings, the European Centre for Disease Prevention and Control published two reports [11,14], and the United States Centers for Diseases Control and Prevention recommended ‘mosquito avoidance precaution’ to travellers to agricultural areas of Ginosa [13].

In this report we describe a cryptic severe *P. falciparum* malaria case observed in a Moroccan person living in Tuscany, Italy.

Morocco was certified as malaria free country in 2010, not reporting autochthonous cases since 2007 [15]. Imported *P. falciparum* malaria cases, mainly from Sub-Saharan Africa, are reported, and a risk of reintroduction is considered [16,17]. Our patient presented symptoms 73 days after leaving Morocco, making it unlikely that the infection was acquired in Morocco since the incubation period for *P. falciparum* ranges in most cases from 9 to 30 days [18]. The possibility that the patient had been infected during a previous 1-month stay in Ghana, in 2014, and had developed the disease after such a long incubation period appears unlikely, although it cannot be completely ruled out, as prolonged incubation has been reported in the recent literature [19]. On rare occasions, cases of *P. falciparum* malaria with delayed onset have been described in semi-immune migrants from endemic areas in which the immunity tends to wane years after migration [20]. The possibility of ‘airport malaria’ could be excluded, since the nearest airports to the Tuscany coast (Pisa and Grosseto) are at least ca 15 km far from the patient visited areas. Other travels to endemic areas and risk factors for induced malaria such as transfusion, transplant, nosocomial, intravenous drug use were denied by the patient. The possibility of transmission by an infected mosquito transported in a baggage cannot be excluded [21].

Malaria competent vectors are present in Italy and, according to the latest available entomological data, *An. labranchiae* is present in Tuscany [10]. The level of competence of *An. labranchiae* to transmit *P. falciparum* is debated. Studies conducted on vectorial capacity of Italian *An. labranchiae* showed that the species is poorly susceptible to infection with tropical or African *P. falciparum* strains [22-24]. Moreover, a study from 2012 assessed the malariogenic potential of the Maremma as very low, although the occurrence of sporadic, isolated cases of introduced *P. vivax* malaria was considered possible during the summer season, in presence of asymptomatic gametocyte carriers from endemic areas [24]. Concerning *P. falciparum*, the estimated vectorial capacity of anopheline population in Maremma is considered even lower [24].

The presented case, together with other *P. falciparum* malaria cases recently reported from Apulia, may suggest the occurrence of sporadic autochthonous *P. falciparum* during the summer season in Italy. However, considering that the susceptibility to *P. falciparum* infection of the anthropophilic *Anopheles* species present in Tuscany area is very low, and that the possibility of unrevealed risk factors for acquiring malaria cannot be completely excluded, the presented case remains cryptic. Some travellers may inadvertently or deliberately fail to mention that they have recently been in an endemic area [25].

The fact that four patients recently diagnosed with *P. falciparum* malaria in Italy were Moroccan citizens remains difficult to explain, considering that Morocco is a malaria-free country. In our patient, malaria diagnosis was delayed because of the missing history of a recent visit to a malaria endemic area. In this perspective, clinicians should consider malaria as one possible differential diagnosis in patients with compatible clinical and laboratory features, even if they do not report a clear exposure to endemic countries.
